# The Effects of Pulmonary Physical Therapy on the Patients with Respiratory Failure

**Published:** 2018-07

**Authors:** Lei GAI, Yan TONG, Biqing YAN

**Affiliations:** Intensive Care Unit, The Affiliated Hospital of Medical Collage, Ningbo University, Ningbo, Zhejiang 315000, P.R. China

**Keywords:** Respiratory failure, Pulmonary physical therapy, Therapeutic effect

## Abstract

**Background:**

We aimed to investigate the application effects of pulmonary physical therapy on the patients with respiratory failure.

**Methods:**

Overall, 132 patients with respiratory failure admitted into the Affiliated Hospital of Medical Collage of Ningbo University from 2013–2017 were enrolled and divided into control group (n=66) and observation group (n=66). Patients in the two groups received conventional physical therapy but those in observation group received pulmonary physical therapy additionally. The ventilation and air-exchanging functions, scores of acute physiology and chronic health evaluation II (APACHE II) and occurrence of complications in patients in the two groups were compared.

**Results:**

PaO2, PaCO2, PaO2/FiO2 and estimated FEV2% were greatly improved in patients in the two groups after treatment and those in patients in observation group were better than those in patients in control group; the differences were statistically significant (*P*=0.014). There were no statistically significant differences in the scores of APACHE II in patients in the two groups before treatment and at 2 days and 3 days after treatment. Scores of APACHE II of patients in observation group were obviously lower than those of other group at 4 days and 7 days after treatment and the differences were statistically significant (*P*=0.015, 0.029). The total incidence rates of complications in patients in observation group and control group were 7.57% and 39.39%, respectively, and the difference was statistically significant (*P*=0.021).

**Conclusion:**

The treatment of respiratory failure patients with pulmonary physical therapy can greatly improve the ventilation and air-exchanging functions, avoid the occurrence of complications and improve the health condition.

## Introduction

Respiratory failure is a severe respiratory dysfunction with complex causes. Patients are usually accompanied by increased PaCO2 and decreased PaO2. Clinically, patients are treated with ventilator therapy in most cases, which can significantly reduce the respiratory power consumption of patients and reduce the risk of treatment (1). However, invasive mechanical ventilation may lead to complications, such as atelectasis and respiratory muscle-related pneumonia, in patients. Deep venous thrombosis (DVT) will occur due to the long-term limited activities of patients (2).

For pulmonary physical therapy, standardized nursing procedure is adopted to maintain normal lung ventilation and air-exchanging functions through physical measures such as assessment of pulmonary conditions, percussion on back, aerosol inhalation, cough movement, vibration row phlegm, postural drainage and sputum suction. It is commonly used in the treatment of patients in intensive care unit (3, 4).

In this study, the application effects of pulmonary physical therapy on patients with respiratory failure were explored in order to provide references for clinical treatment in the later period. Now it is analyzed and reported as follows.

## Materials and Methods

### General data

A total of 132 patients with respiratory failure admitted into the Affiliated Hospital of Medical Collage of Ningbo University from January 2013 to January 2017 were selected as subjects. There were 66 patients in observation group including 47 males and 19 females who were aged 18 to 71 years old with an average age of (62.18±4.28) yr old. There were 66 patients in control group including 45 males and 21 females who were aged 19 to 70 yr old with an average age of (61.77±5.03) yr old. There were no statistically significant differences in the general data of patients in the two groups. Therefore, they were comparable.

The study was approved by the Ethics Committee of the Affiliated Hospital of Medical Collage of Ningbo University.

Inclusion criteria: 1) Patients treated with invasive mechanical ventilation for ≥ 48 h; 2) patients aged ≥ 18 yr old; 3) patients with endotracheal tube indwelled through mouth; 4) patients with good pulmonary function; 5) patients who were volunteered to participate in the study and signed the informed consent.

Exclusion criteria: 1)Patients with contraindications of pulmonary physical therapy; 2) patients with sternal fracture or rib fracture; 3) patients complicated by dysfunction of bleeding or coagulation; 4) patients who gave up halfway or died; 5) patients with severe mental disorder.

### Methods

Patients in the two groups were all given conventional physical managements such as percussion on back, roll over and postural drainage. Patients in observation group were treated with pulmonary physical therapy additionally: 1) Qualified patients were given pulmonary physical treatment after the assessment of the disease condition, contraindication and indication of patients; 2) Manipulative lung inflation: Patients were treated with manipulative lung inflation 2 times/day. The artificial breathing bag with oxygen storage device was connected to the oxygen with a flow of 8 L/min to 10 L/min. The tidal volume was made 1.5 times larger than that of the normal volume; squeezing frequency was 10–12 times/min; breath of patients was held for 2 s after the end of aspiration of air. The artificial breathing bag was released quickly on expiration to produce pressure difference inside and outside the airway, so as to exclude the airway secretion; 3) Vibration: Vibratory sputum elimination machine was used for patients 2 times/day for 15–20 min per time with a frequency of 20–30 cps; 4) Early function rehabilitation: Patients were engaged in the active and passive limb movements, including fist exercise, arm lifting exercise, flexion and extension exercises of lower extremity knee and hip, ankle pump exercise, breathing exercise and expectoration once a day and 15–30 min each time.

The study was approved by the Ethics Committee of the Affiliated Hospital of Medical Collage of Ningbo University.

### Observation indexes

The ventilation and air-exchanging functions, acute physiology and chronic health evaluation scoring system II (APACHE II) scores at different time points and the occurrence of complications in patients in the two groups were compared. 1) Arterial oxygen partial pressure (PaO2), arterial partial pressure of carbon dioxide (PaCo2), oxygenation index (PaO2/FiO2) and lung function (estimated FEV2%) in patients were monitored; 2) the APACHE II scores of patients were monitored before treatment, and at 2 days, 3 days, 4 days and 7 days after treatment; 3) complications: including the ventilator-related pneumonia, pulmonary atelectasis, pulmonary edema and deep venous thrombosis (DVT).

### Statistical methods

Statistical Product and Service Solutions (SPSS) 20.0 (Chicago, IL, USA) was applied for the statistical analysis. The measurement data and counting data were determined through *t* test and *x*^2^ test. *P*<0.05 suggested that the difference was statistically significant.

## Results

### Comparisons of ventilation and air-exchanging functions

The differences in PaO2, PaCO2, PaO2/FiO2 and estimated FEV2% in patients in the two groups were not statistically significant before treatment (*P*=0.065, 0.087, 0.077, 0.131). Compared with those in patients before treatment, PaO2, PaCO2, PaO2/FiO2 and estimated FEV2% in patients after treatment were greatly improved. Those in patients in observation group were superior to those in patients in control group and the differences were statistically significant (*P*=0.014, 0.045, 0.035, 0.020) (Table 1).

**Table 1:** Comparisons of ventilation and air exchanging functions

***Group***	***PaO2 (mmHg)***		***PaCO2 (mmHg)***		***PaO2/FiO2***		***Estimated FEV2%***	
	***Before treatment***	***After treatment***	***Before treatment***	***After treatment***	***Before treatment***	***After treatment***	***Before treatment***	***After treatment***
Observation group (n=66)	49.94±13.21	78.39±8.95	75.34±11.93	63.17±9.55	238.53±22.44	316.28±9.63	49.34±4.24	76.59±5.22
Control group (n=66)	48.68±12.28	63.54±9.04	77.27±10.71	71.08±9.13	243.26±23.45	281.78±9.71	50.38±4.38	62.69±4.71
*t* value	1.921	2.559	1.693	2.013	1.813	2.317	1.629	2.511
*P* value	0.065	0.014	0.087	0.045	0.077	0.035	0.131	0.020

### Comparisons of APACHE II scores at different time points

The differences in the scores of APACHE II of patients in the two groups were not statistically significant before treatment, and at 2 days and 3 days after treatment (*P*=0.079, 0.068, 0.054). APACHE II scores of patients in observation group were obviously lower than those of patients in control group at 4 days and 7 days after treatment and the differences were statistically significant (*P*=0.015, 0.029) (Table 2).

**Table 2:** Comparisons of APACHE II scores at different time points

***Group***	***APACHE II score***				
	***Before treatment***	***2 d after treatment***	***3 d after treatment***	***4 d after treatment***	***7 d after treatment***
Observation group (n=66)	18.34±5.37	14.08±5.12	12.44±5.02	8.70±4.55	7.71±5.03
Control group (n=66)	18.16±5.16	13.72±4.88	12.31±4.75	12.01±5.32	11.78±4.87
*t* value	1.783	1.912	1.950	2.557	2.435
*P* value	0.079	0.068	0.054	0.015	0.029

### Comparisons of complications

The overall incidence rates of complications in patients in observation group and control group were 7.57% and 39.39%, respectively, and the difference was statistically significant (*P*=0.021) (Table 3).

**Table 3:** Comparisons of complications [n (%)]

***Group***	***Ventilator-related pneumonia***	***Pulmonary atelectasis***	***Pulmonary edema***	***DVT***	***Overall incidence rate***
Observation group (n=66)	4 (6.06)	0 (0.00)	1 (1.51)	0 (0.00)	5 (7.57)
Control group (n=66)	16 (24.24)	6 (9.09)	2 (3.03)	2 (3.03)	26 (39.39)
*t* value					6.921
*P* value					0.021

## Discussion

Pulmonary physical therapy is a treatment method that combines the physiological characteristics and physical principles of body for the treatment and prevention of lung diseases, which drains out the respiratory secretions with better effects based on the principle of physical methods to ensure a clear airway and maintain the normal state of respiratory tract (5). There is a physical nursing mode during the whole process from the secretion of sputum to the expectoration out the airway in this therapy. During the sputum secretion process, sputum in the respiratory tract can be loosened through the aerosol inhalation, postural conversion and chest wall vibration to reduce the viscosity of sputum. At the same time, vibration outside the chest wall, lung inflation and other techniques also can strengthen cough and help eliminate the sputum (6, 7). The clinical treatment of respiratory failure, as a complex physiological disorder, needs to ensure that the respiratory tract is clear and water-electrolytes are stable. Therefore, the application effects of pulmonary physical therapy on the patients with respiratory failure were explored in this study, in order to provide references for the clinical treatment in later period.

The results of this study showed that the PaO2, PaCO2, PaO2/FiO2 and estimated FEV2% in patients in the two groups were significantly improved after treatment compared with those before treatment. The above values in patients in observation group were superior to those in patients in control group and the differences were statistically significant (*P*=0.065, 0.087, 0.077, 0.131), indicating that the ventilation and air-exchange functions of patients in observation group were improved greatly. This is mainly because in the pulmonary physical therapy, the pulmonary conditions are evaluated, and the means including postural drainage, breathing exercise, pulmonary dilation, vibration and tapping, kinesitherapy and airway suction are used to help secretions in the bronchium and pulmonary alveoli enter into the large bronchus, improve oxygenation and ventilation, improve the pulmonary compliance, promote the lung inflation and ensure the clear trachea (8).

Meanwhile, it was found in this study that the incidence rates of complications in patients in observation group and control group were 7.57% and 39.39%, respectively, and the difference was statistically significant (*P*=0.021). This may be because the pulmonary physical therapy can improve the success rate of ventilator weaning for patients with respiratory failure, and shorten the weaning time, thereby reducing the incidence rate of ventilator-related complications (9).

Clinically, APACHE II score is now widely used in intensive care unit and in the most authoritative critical illness assessment system, which can determine the condition of patients with severe illness. It also can conduct the objective prediction of mortality of those patients. This can be taken as a basis to help physicians revise the care plan in a timely manner and use the medical resources rationally, thus improving the quality of care (10). Our study reported that there were no significant differences in APACHE II scores between patients in the two groups before treatment and at 2 days and 3 days after treatment (*P*=0.079,0.068,0.054). The APACHE II scores in observation group at 4 days and 7 days after treatment were significantly lower than those in control group (*P*=0.015,0.029). This is because patients can not only prevent complications through the pulmonary physical therapy, but also prevent muscle failure to help improve the daily living ability, and reduce the incidence rate of DVT through early function rehabilitation, thereby improving APACHE II scores (11).

However, in clinical practice, it was found that if the sputum of patients is viscous, the sputum can be diluted by aerosol inhalation. After patients moisturize throat with a proper amount of water, the pulmonary physical therapy can achieve a better effect. Patients need to take the sitting or semi-recumbent position in the process of chest vibration or tapping, and the lateral position or supine position for postural drainage, instead of head-down position. Health care workers should be patient to explain the work before treatment and perform the treatment step by step, also closely monitor the breath, blood pressure, oxygen saturation and pulse of patients and stop the treatment immediately once the patient is discomfort (12).

## Conclusion

The pulmonary physical therapy can significantly improve the ventilation and air-exchanging functions of patients with respiratory failure, prevent the occurrence of complications and improve the state of health, which can be further promoted and used clinically.

## Ethical considerations

Ethical issues (Including plagiarism, informed consent, misconduct, data fabrication and/or falsification, double publication and/or submission, redundancy, etc.) have been completely observed by the authors.
